# A patient with a history of breast cancer and multiple bone lesions: a case report

**DOI:** 10.1186/s13256-017-1296-1

**Published:** 2017-05-06

**Authors:** Marie-Angela Schnyder, Paul Stolzmann, Gerhard Frank Huber, Christoph Schmid

**Affiliations:** 1 Division of Endocrinology, Diabetes and Clinical Nutrition, University Hospital Zurich, Rämistrasse 100, 8091 Zurich, Switzerland; 2 Department of Nuclear Medicine, University Hospital Zurich, Rämistrasse 100, 8091 Zurich, Switzerland; 3 Department of Otorhinolaryngology, Head and Neck Surgery, University Hospital Zurich, Rämistrasse 100, 8091 Zurich, Switzerland

**Keywords:** Hypercalcemia, Primary hyperparathyroidism, Osteitis fibrosa cystica, Brown tumors, Hungry bone syndrome, ^18^F-FDG-PET, Lytic bone lesions

## Abstract

**Background:**

Long-term severe hyperparathyroidism leads to thinning of cortical bone and cystic bone defects referred to as *osteitis fibrosa cystica*. Cysts filled with hemosiderin deposits may appear colored as “brown tumors.” Osteitis fibrosa cystica and brown tumors are occasionally visualized as multiple, potentially corticalis-disrupting bone lesions mimicking metastases by bone scintigraphy or ^18^F-fluorodeoxyglucose positron emission tomography.

**Case presentation:**

We report a case of a 72-year-old white woman who presented with malaise, weight loss, and hypercalcemia. She had a history of breast cancer 7 years before. The practitioner, suspecting bone metastases, initiated bone scintigraphy, which showed multiple bone lesions, and referred her to our hospital for further investigations. Laboratory investigations confirmed hypercalcemia but revealed a constellation of primary hyperparathyroidism and not hypercalcemia of malignancy; in the latter condition, a suppressed rather than an increased value of parathyroid hormone would have been expected. A parathyroid adenoma was found and surgically removed. The patient’s postoperative course showed a hungry bone syndrome, and brown tumors were suspected. With the background of a previous breast cancer and lytic, partly corticalis-disrupting bone lesions, there was a great concern not to miss a concomitant malignant disease. Biopsies were not diagnostic for either malignancy or brown tumor. Six months after the patient’s neck surgery, imaging showed healing of the bone lesions, and bone metastases could be excluded.

**Conclusions:**

This case shows essential differential diagnosis in a patient with hypercalcemia and multiple bone lesions. Whenever multiple, fluorodeoxyglucose-avid bone lesions are found, malignancy and metabolic bone disease should both be included in the differential diagnosis. Fluorodeoxyglucose-avid and corticalis-disrupting lytic lesions also occur in benign bone disease. There may be very few similar cases with heterogeneous and widespread bone lesions reported in the literature, but we think our patient’s case is particularly remarkable for its detailed imaging and the well-documented course.

## Background

Osteitis fibrosa cystica (OFC) is a rare skeletal complication of long-term severe hyperparathyroidism (HPT) [1]. Increased bone remodeling resulting from longer-term excessive stimulation of osteoclasts by parathyroid hormone (PTH) leads to subperiosteal resorption, thinning of cortical bone, and occasionally cystic defects. The latter can be filled with hemosiderin, deposits visualized as brown-colored “tumors” [2]. These focal reactive lesions can be found in patients with primary or secondary HPT. In developed countries, OFC and brown tumors (BTs) became rare as a result of the wide application of routine calcium screening and increased medical attention [3, 4]. BTs are often multiple, fluorodeoxyglucose (FDG)-avid, potentially corticalis-disrupting bone lesions mimicking metastases on bone scintigraphy or ^18^F-FDG-positron emission tomography (PET) [2, 4–6]. Serum calcium and PTH measurements are excellent diagnostic tools, whereas bone biopsy may be less informative but is occasionally performed to rule out an accompanying malignancy [3]. Parathyroidectomy (PTX) is the treatment of choice in severe HPT.

## Case presentation

**Fig. 1:**
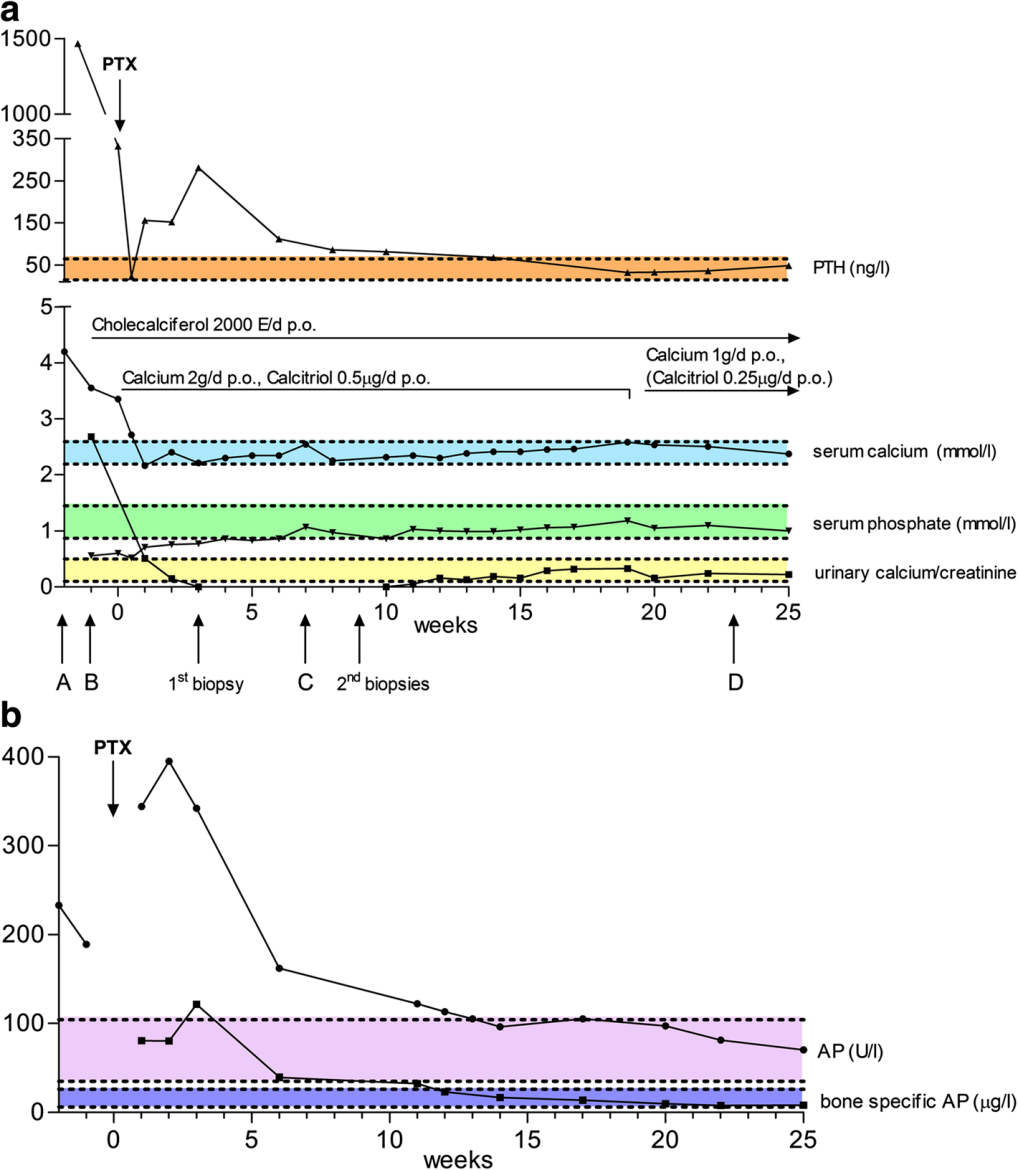
**a** Parathyroid hormone (PTH), serum calcium, serum phosphate, and calciuria time course. Time 0 is defined as the day of parathyroidectomy (PTX). The *dashed lines* and *colors* show the lower and upper limits of parathyroid hormone (15–65 ng/L) in *orange*, albumin-corrected serum calcium (2.19–2.59 mmol/L) in *blue*, serum phosphate (0.87–1.45 mmol/L) in *green*, and calcium-to-creatinine molar ratio in urine (0.1–0.5) in *yellow*. Parathyroid hormone, calcium, and calciuria are dramatically increased at baseline, and phosphate is low. Vitamin D_3_ (cholecalciferol) was started at week −1. Calcium therapy was conducted orally (2 g/day for weeks 0–19, then reduced to 1 g/day). Calcitriol 0.5 μg/day was given from weeks 0 to 19, then reduced to 0.25 μg/day and stopped at week 25. The *capital letters* and *arrows* indicate the time points at which imaging (Fig. 2a–d) and biopsies, respectively, were performed. **b** Alkaline phosphatase (AP; total activity) and bone-specific alkaline phosphatase mass time course. The *dashed lines* and *colors* show the lower and upper limits of alkaline phosphatase (35–104 U/L) in *bright purple* and bone-specific alkaline phosphatase (postmenopausal; 6–26 μg/L) in *dark purple*. Alkaline phosphatase was high before parathyroidectomy (reflecting high bone turnover resulting from hyperparathyroidism) and further increased (reflecting increased bone formation [hungry bone syndrome]) after parathyroidectomy

**Fig. 2:**
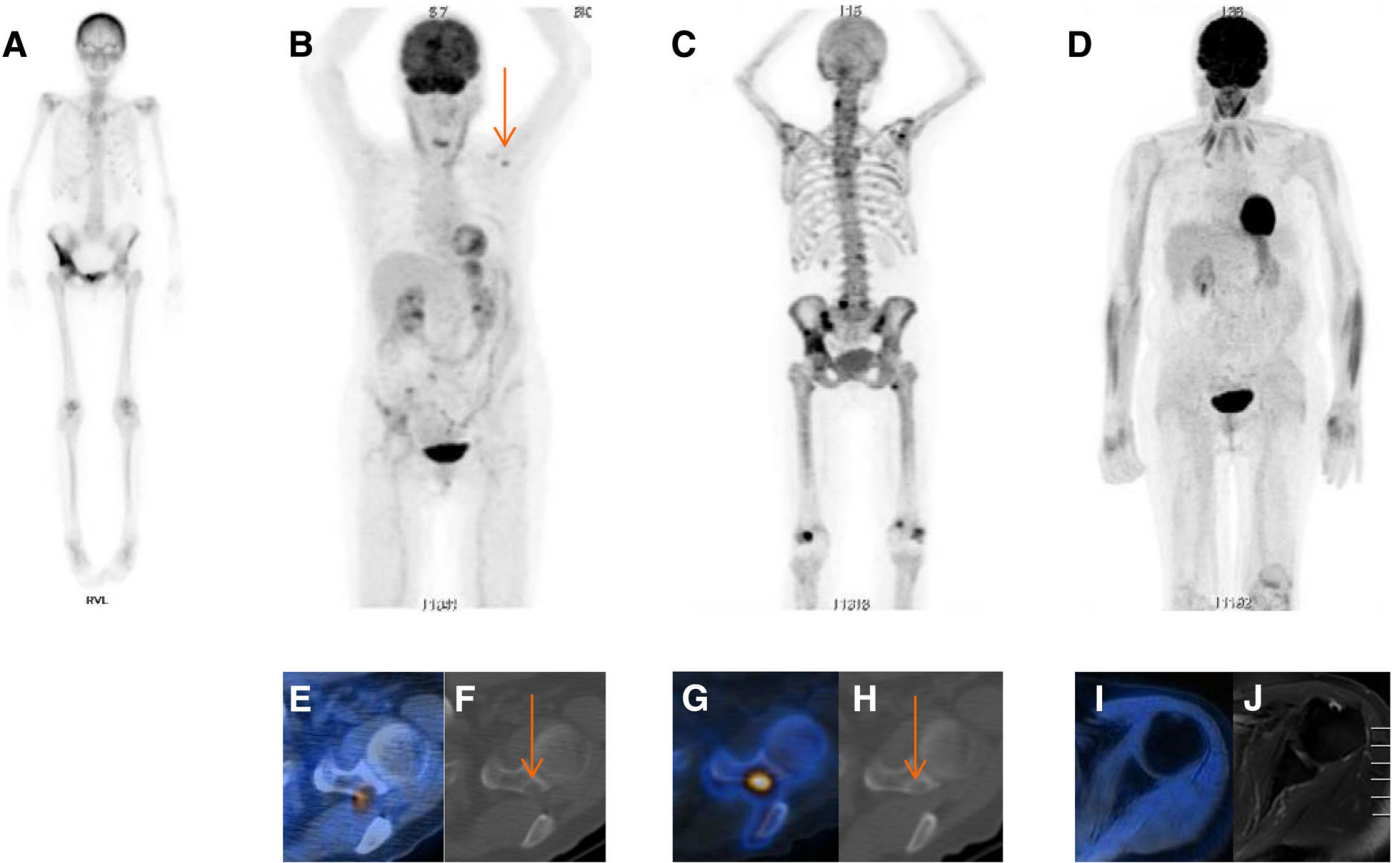
Radiographic imaging. Timing is indicated by *arrows* with *capital letters* in Fig. 1a. **a** Technetium-99m-3,3-diphosphono-1,2-propanodicarboxylic acid scintigraphy with multiple hot lesions in the os ilium and acetabulum on the right-hand side. Note diffuse uptake in the calvarium and focally pronounced uptake at the tip of the ribs, suggestive of hyperparathyroidism. **b** ^18^F-Fluorodeoxyglucose positron emission tomography demonstrates multiple metabolically active bone lesions in correspondence with technetium-99m-3,3-diphosphono-1,2-propanodicarboxylic acid scan, but additional fluorodeoxyglucose-positive lesions were detected, such as in the glenoid on the left-hand side (*arrow*). **c** On this ^18^F-fluoride-positron emission tomographic scan, all lesions demonstrate fluoride uptake as proof of mineralization. Note fluoride uptake in flat bones and in costochondral junctions (similarly to technetium-99m-3,3-diphosphono-1,2-propanodicarboxylic acid whole-body scintigraphy) thought to be indicative of hyperparathyroidism and brown tumors. **d** Follow-up ^18^F-fluorodeoxyglucose positron emission tomography with no fluorodeoxyglucose-avid lesions demonstrating a complete response 23 weeks after parathyroidectomy. Initial ^18^F-fluorodeoxyglucose positron emission tomography/computed tomography shows an additional fluorodeoxyglucose-positive lesion in the left glenoid (**e**) not depicted in former technetium-99m-3,3-diphosphono-1,2-propanodicarboxylic acid scanning. The lesion exhibits sharply demarcated borders and evidence of subperiosteal bone resorption on computed tomography (**f**, *arrow*) thought to be pathognomonic for hyperparathyroidism. The lesion shows fluoride uptake in ^18^F-fluoride positron emission tomography/computed tomography (**g**) and demonstrates progressive sclerosis of the central matrix in computed tomography (**h**, *arrow*) after initiation of therapy. In ^18^F-fluorodeoxyglucose positron emission tomography/magnetic resonance imaging 23 weeks after parathyroidectomy, neither abnormal fluorodeoxyglucose uptake nor signal abnormalities are detected in the glenoid in T1-weighted (**i**) and T2-weighted images (**j**)

A 72-year-old white woman was admitted to our hospital because of newly diagnosed hypercalcemia (albumin-corrected calcium 4.2 mmol/L; normal range 2.19–2.59 mmol/L) (Fig. 1a) and multiple bone lesions visualized by bone scintigraphy. For 6 months, she had experienced progressive weakness, bone pain, nausea, and body weight loss of 4 kg. In addition, she noticed nocturia and polydipsia. According to her husband, she had recently become depressive and forgetful. Seven years before, she had been treated for breast cancer. The tumor stage was pT1c, pN0, M0, G2, R0; her estrogen and progesterone receptor status was positive; and she was herceptin 2 receptor-negative. At that time, a segment resection and a sentinel lymph node biopsy, as well as radio- and chemotherapy, were carried out. No local tumor recurrence had been detected in the last ultrasonographic follow-up of the breast and lymph nodes a few months earlier. Having found hypercalcemia and an elevated alkaline phosphatase (AP) level (233 U/L; normal range 35–104 U/L), her practitioner directly initiated whole-body bone technetium-99m-3,3-diphosphono-1,2-propanodicarboxylic acid (^99m^Tc-DPD) scintigraphy (Fig. 2a), which showed multiple active hits, with the biggest lesions located in the os ilium and acetabulum on the right-hand side; unspecific, diffuse uptake in the calvarium; and multiple sites of focal uptake at costochondral junctions. In the context of the patient’s history of breast cancer, she was suspected of having multiple bone metastases.

On examination, the patient appeared to be in an afebrile, hemodynamically stable state, and no neurologic deficits were noticed. Her body mass index was 20.5 kg/m^2^. Laboratory studies showed hypercalcemia (albumin-corrected calcium 3.55 mmol/L), low phosphate (0.56 mmol/L; normal range 0.87–1.45 mmol/L), impaired renal function (creatinine 119 μmol/L, estimated glomerular filtration rate of 39 ml/minute), elevated AP (189 U/L), and a grossly elevated PTH level (1466 ng/L; normal range 15–65 ng/L) (Fig. 1a, b). 25-OH-vitamin D was slightly decreased (14.2 μg/L, normal >20 μg/L). Tumor marker CA15-3 was normal (26.7 kU/L, normal <30 kU/L).

On the basis of the laboratory constellation of primary HPT, neck surgery was considered and proposed to the patient. We felt that this patient could benefit from parathyroid surgery and correction of hypercalcemia, regardless of a potential concomitant malignancy. Sonography and scintigraphy of the parathyroid glands were performed, and a parathyroid adenoma (>2 cm in diameter) was detected at the left inferior side. Concerning the possible metastatic bone disease, ^18^F-FDG-PET/computed tomography (CT) was carried out (Fig. 2b). This examination provided evidence of multiple metabolically active, predominantly lytic bone lesions in the pelvis and at the sternum as well as a corticalis-disrupting lesion in the glenoid on the left-hand side (Fig. 2e, f).

The patient received intravenous continuous hydration (isotonic saline) and oral vitamin D_3_ (cholecalciferol 2000 U/day), and she consented to the proposed parathyroid surgery, which was carried out 1 week later. Following removal of the enlarged parathyroid gland (suspected to be the single source of excessive PTH; weighing 4.5 g), the patient’s intraoperative PTH level decreased to 22.6 ng/L (Fig. 1a), indicating removal of the culprit lesion. As expected, the lesion was posterior to the left lower thyroid pole and completely removed. In the postoperative course, no local (neck) complications were noticed.

After surgery, there was a progressive fall in serum calcium (total calcium down to a nadir of 1.96 mmol/L on day 5; albumin-corrected as shown in Fig. 1a), whereas phosphate remained low (0.52 mmol/L on the fifth postoperative day, returning to within the normal range 7 weeks later), and AP activity further increased up to 395 U/L 2 weeks after PTX (Fig. 1b). This increase could be attributed to an increase in the bone isoenzyme because bone-specific AP mass (up to 121 μg/L, normal postmenopausal range 6–26 μg/L) peaked at around the same time and came back to within the normal range by week 12 after PTX. PTH increased during the initial postoperative course (with low postoperative calcium) to 300 ng/L, ruling out hypoparathyroidism, and fasting urinary spot calcium excretion decreased to a level below the detection limit (Fig. 1a) and remained low (urinary calcium/creatinine molar ratio <0.1) until 10 weeks after surgery. This constellation suggested hungry bone syndrome (HBS), a common consequence following successful parathyroid surgery in patients with OFC, and we considered this postoperative course compatible with the bone lesions [1]. We started therapy with oral calcium (2 g/day) and calcitriol (0.5 μg/day), we continued treating the patient with vitamin D_3_ (2000 U/day, long-term). Owing to initial diagnostic uncertainty, signals sent by some physicians to the patient, and her fear of recurring malignancy, the largest lesion in the area of the pelvis was biopsied with CT guidance in an outpatient setting (3 weeks after PTX) (Fig. 1a). No malignant cells could be found, but the material turned out to be insufficient for pathological diagnosis by histological and immunohistochemical investigations. With the aim of gaining more information in the setting of an elevated AP (reflecting elevated bone turnover), we performed ^18^F-fluoride-PET (Fig. 2c, g, h) 7 weeks after PTX. In this investigation, all the lesions showed increased fluoride uptake and progressive sclerosis of some lesions reflecting bone mineralization. Additional open biopsies were performed 9 weeks after PTX (Fig. 1a). Tissue samples of the right patella and of the right os ilium (the most reachable lesions) were obtained; again, no malignant cells were found.

We regularly saw the patient in our consultation, and laboratory controls showed that the serum calcium could be kept in the normal range over weeks (Fig. 1a). The therapy with calcium and calcitriol was adapted continuously. Six months after PTX, the patient presented with a joint effusion of the left knee. The rheumatologists conducted a joint puncture, and aspiration material showed calcium pyrophosphate crystals. Twenty-three weeks after PTX and bone lesions of unknown etiology and significance, we conducted further follow-up imaging by ^18^F-FDG-PET/magnetic resonance imaging (MRI) (Fig. 2d, i, j) to prove BTs and again rule out malignancy. The bone lesions were no longer detectable in the PET component of the examination. One year after PTX, the patient no longer had joint pain, remained normocalcemic, was physically active, and found herself, in retrospect, much stronger than in many years before.

## Discussion

Our patient presented with characteristic symptoms and signs of HPT. She experienced upper abdominal pain; polyuria (caused by an acquired resistance to antidiuretic hormone in the setting of hypercalcemia); polydipsia; fatigue; and, as we learned later, nephrolithiasis a few years before. Weight loss and bone pain were initially misleading and interpreted as red flags of tumor disease. The occurrence of chondrocalcinosis (occasionally termed *pseudogout*, currently termed *calcium pyrophosphate deposition disease*) a few months after PTX is an entity that has been described in many cases but is not widely recognized in clinical practice [7, 8].

The laboratory course with postoperative hypocalcaemia, disappearance of calcium from urine samples (calciuria initially below the limit of detection), rise of AP (but not of phosphate), and an appropriate response of the remaining parathyroids to serum calcium changes is typical for HBS, which also supported the diagnosis of OFC and BTs [1]. A low phosphate level (<0.97 mmol/L) in conjunction with a low calcium level (<2.1 mmol/L) a few days after PTX for HPT has been used to define HBS by some authors [9]. However, HBS could be considered as mild in our patient because oral treatment turned out to be sufficient to maintain calcemia and to prevent tetany, and the patient could be discharged from the hospital 8 days after PTX.

Histopathology is commonly considered the gold standard for medical diagnosis, especially in oncology. Biopsies are therefore frequently recommended. In patients with HPT and OFC, typical histopathological findings of BT biopsies may be hemosiderin deposits, increased osteoclasts, mononuclear cells, and fibroblasts [2, 10, 11]. However, as in our patient, biopsies often show unspecific characteristics and thus remain nondiagnostic, also owing to the small sample sizes.

In general, BTs present as single or multiple lesions. Common sites are flat bones, such as the maxillofacial bone, ribs, clavicle, and pelvic girdle, as well as long bones, with the diaphysis most commonly affected (where BTs most likely cause symptoms) [3, 10]. In keeping with this observation, our patient’s lesions were situated predominantly in the patellae, pelvic alae, and scapula. The lesion in the left glenoid demonstrated subperiosteal bone resorption (Fig. 2e, f), which is suggestive of BT. Metastatic involvement is related to skeletal blood flow and thus may occur predominantly in the axial skeleton, but it can be scattered throughout the skeleton [3].

FDG-positive lesions in PET examinations always raise suspicion of malignancy because cancers are expected to have high glucose requirements for growth and their metabolism. However, there remain benign differential diagnoses; for example, inflammatory cells may show increased metabolic activity. BTs can present with FDG avidity in PET despite having benign characteristics [5, 6]. In our patient, there was a picture of multiple, not uniformly distributed (active and inactive) bone lesions in ^18^F-FDG-PET (Fig. 2b). ^18^F-fluoride-PET (Fig. 2c) is a very sensitive but unspecific method to detect loci of increased bone remodeling. Because the patient had undergone PTX but no chemotherapy before, the progressive calcification of the bone lesions was more likely encouraging the diagnosis of BTs rather than metastases. Of note, blastic bony lesions are considered typical of metastatic breast cancer; however, lytic metastases do occur. Regarding our experience, we think that progressive mineralization of formerly lytic bone metastases occurs only as a response to antitumor treatment, which was not given to our patient. In general, whole-body bone scintigraphy can evaluate patients for skeletal metastases, and ^18^F-FDG-PET seems to exhibit high specificity and accuracy in detecting bone metastases. However, there is a limitation of ^18^F-FDG-PET in assessing initially sclerotic bone metastases because some lesions may go undetected, so that ^18^F-fluoride-PET might be useful [12].

The key treatment of OFC is PTX. The course of the disease often takes several months, and the adequate substitution of calcium up to 1 year after parathyroid surgery is important to support bone recovery. After 6 months, we performed another radiographic control because of a persistent concern about missing a concomitant malignant disease. Thus, the patient underwent follow-up ^18^F-FDG-PET/MRI (Fig. 2d) a few months after PTX when her bones were no longer too “hungry.” This scan provided evidence to rule out recurrent breast cancer. We chose PET/MRI (3.4 mSv) over PET/CT to keep the patient’s radiation exposure as low as reasonably achievable by omitting the additional CT scan.

## Conclusions

In our patient’s case, combining information concerning the clinical presentation and course, the biochemistry laboratory examinations, and the imaging (including functional and PET imaging) turned out to be more useful than biopsies and histopathology. In the overall context, we finally made the diagnosis of BTs caused by severe primary HPT.
